# Reliability and Reproducibility of Landmark Identification in Unilateral Cleft Lip and Palate Patients: Digital Lateral Vis-A-Vis CBCT-Derived 3D Cephalograms

**DOI:** 10.3390/jcm10030535

**Published:** 2021-02-02

**Authors:** Anuraj Singh Kochhar, Ludovica Nucci, Maninder Singh Sidhu, Mona Prabhakar, Vincenzo Grassia, Letizia Perillo, Gulsheen Kaur Kochhar, Ritasha Bhasin, Himanshu Dadlani, Fabrizia d’Apuzzo

**Affiliations:** 1 Former Consultant Orthodontist Max Hospital Gurgaon, Haryana 122001, India; 2 Multidisciplinary Department of Medical-Surgical and Dental Specialties, University of Campania Luigi Vanvitelli, 80138 Naples, Italy; ludovica.nucci@unicampania.it (L.N.); grassiavincenzo@libero.it (V.G.); letizia.perillo@unicampania.it (L.P.); fabrizia.dapuzzo@unicampania.it (F.d.); 3 Department of Orthodontics & Dean, Research & Development Faculty of Dental Sciences, SGT University Gurugram, Haryana 122505, India; deanresearch@sgtuniversity.org (M.S.S.); mona.prabhakar@sgtuniversity.org (M.P.); 4 Department of Pediatric & Preventive Dentistry, National Dental College & Hospital, Punjab 140507, India; gulsheenuppal@gmail.com; 5 Faculty of Dentistry, University of Toronto, Toronto, ON M5G1G6, Canada; ritasha.bhasin@mail.utoronto.ca; 6 Senior Consultant Department of Dentistry (Periodontology), Max Hospital, Gurgaon, Haryana 122001, India; himdent@hotmail.com

**Keywords:** cleft, accuracy, unilateral cleft lip and palate, CBCT, interobserver, cephalogram

## Abstract

Background: The aim of the retrospective observational study was to compare the precision of landmark identification and its reproducibility using cone beam computed tomography-derived 3D cephalograms and digital lateral cephalograms in unilateral cleft lip and palate patients. Methods: Cephalograms of thirty-one (31) North Indian children (18 boys and 13 girls) with a unilateral cleft lip and palate, who were recommended for orthodontic treatment, were selected. After a thorough analysis of peer-reviewed articles, 20 difficult-to-trace landmarks were selected, and their reliability and reproducibility were studied. These were subjected to landmark identification to evaluate interobserver variability; the coordinates for each point were traced separately by three different orthodontists (OB_A_, OB_B_, OB_C_). Statistical analysis was performed using descriptive and inferential statistics with paired *t*-tests to compare the differences measured by the two methods. Real-scale data are presented in mean ± SD. A *p*-value less than 0.05 was considered as significant at a 95% confidence level. Results: When comparing, the plotting of points posterior nasal spine (PNS) (*p* < 0.05), anterior nasal spine (ANS) (*p* < 0.01), upper 1 root tip (*p* < 0.05), lower 1 root tip (*p* < 0.05), malare (*p* < 0.05), pyriforme (*p* < 0.05), porion (*p* < 0.01), and basion (*p* < 0.05) was statistically significant. Conclusion: In patients with a cleft lip and palate, the interobserver identification of cephalometric landmarks was significantly more precise and reproducible with cone beam computed tomography -derived cephalograms vis-a-vis digital lateral cephalograms.

## 1. Introduction

Since its advent in 1931 [1], conventional cephalometry has been one of the essential diagnostic tools for analyzing orthodontic problems, maxillofacial deformities, evaluating growth, and planning treatment [2]. However, difficulty in locating imperceivable anatomic landmarks and superimposition of craniofacial structures, owing to the two-dimensional (2D) representation of three-dimensional (3D) anatomy, are amongst the limitations of traditional cephalometrics [3,4,5]. Young cleft lip and palate (CLP) patients usually present with a flattened cranial base, midface deficiency with a retruded maxilla and elongated mandible, anterior and/or posterior crossbites, and an increased vertical dimension, thereby making the recognition of cephalometric landmarks even more arduous [4,5].

The localization of landmarks like point A, the anterior nasal spine (ANS), and the posterior nasal spine (PNS) is challenging due to the decreased opacity resulting from the cleft [6]. The remodeling of the tooth germs in young patients, especially in the anterior contour of the maxilla, can be a possible cause for the same according to Hotz and Gnoinski [7]. According to published data, it is difficult to interpret radiographs in patients with CLP before the exfoliation of the incisors for the abovementioned problems, thus questioning the reliability of cephalometric measurements for the same [8,9].

In the present times, when imaging has been revolutionized, cone beam computed tomography (CBCT) aids in avoiding the anatomic superimpositions and problems caused by magnification, thereby permitting the evaluation of the craniofacial structures from unobstructed perspectives with minimal distortion. Three-dimensional (3D) images from CBCT scans can be rotated easily by changing the rotational axis, as convenient to the observer [2,10]. Specifically, regarding cleft patients, previous studies have advocated CBCT as an exceptional tool for the determination of bone volume [11,12,13,14], bone and root morphology, the assessment of tooth development in the vicinity of the cleft area [15,16,17,18], and soft tissue depth.

In orthodontics, cephalograms are repeatedly traced and reviewed. For impeccable treatment planning, the reliability and reproducibility of these anatomical landmarks are imperative, especially in patients with CLP where the identification of landmarks is highly challenging and, therefore, difficult to perceive and reproduce. Although the synchronal literature in the past several years has emphasized the pivotal role of CBCT to determine the reliability [19,20,21,22,23,24,25,26,27] of anatomic landmarks, there is a paucity of studies for the same, along with their reproducibility in CLP patients versus cephalometrics where the localization of anatomy is challenging.

A lot of studies have emphasized the difficulty in the plotting of bilateral landmarks [2,19,20,21], but none have been done in a compromised craniofacial morphology. To know the actual degree of midfacial retrusion along with the compromised craniofacial morphology that is usually present in cleft patients, the accuracy of landmark identification plays an important role [6,26].

Therefore, the present study was conducted to compare the reliability and reproducibility of landmark identification using two systems: CBCT-derived 3D cephalograms vis-a-vis digital lateral cephalograms in unilateral cleft lip and palate (UCLP) patients.

## 2. Materials and Methods

### 2.1. Ethical Approval

Approval for the retrospective observational study was obtained from Shree Guru Gobind Singh Tricentenary (SGT) Dental College, Hospital, and Research Institute, Budhera, Gurgaon, India (SGTDC/PPL/Com./E.C./14 August 2010) (institutional ethical committee). All records acquired from the Department of Orthodontics and Dentofacial Orthopedics from March 2011 to May 2013 were used only for research purposes, for which prior informed consent had been taken.

### 2.2. Methodology

Examination of records of 54 North Indian children (aged 10–14 years) with a repaired CL ± P anomaly was done. For the sample size estimation in the present study, power analysis at 80% power, a 0.5 alpha level, and an effect size of 0.8 suggested that a minimum of 21 patients were required. Records of thirty-one children (Table 1) who had primary CLP repair performed before 18 months of age, with no secondary alveolar bone grafts and no prior orthodontic/orthopedic appliance intervention (but recommended for comprehensive orthodontic treatment), were selected and designated as samples. Patients’ records not fulfilling any of the above inclusion criteria or with any syndrome or mental retardation as documented in the medical history were excluded. 

Cephalograms had been acquired using PlanmecaPromax (Planmeca Co, Helsinki, Finland) in a natural head position, stabilized by ear rods. The scans were obtained at 66 kvp and 5 mA. The JPG images were obtained and transferred to the Nemoceph NX software (Visiodent, Saint-Denis, France) for calibration and analysis, which was done with a scale present on the X-ray as a 10 mm measurement marked on the cephalogram.

CBCT scans had been acquired using an i-CAT next-generation machine (Imaging Sciences International, Hatfield, PA, USA) with a field of view (fov) of 17 × 22 cm and a scan time of 26 s. The data gathered were saved in DICOM (version 1.7) format with an isometric voxel size of 0.25 mm. The DICOM images, using InVivoDental 5.0 (Anatomage, anatomy imaging software, San Jose, CA, USA), were reoriented according to Kochhar et al. [25]. According to a recent study, the precision of landmark plotting is negligibly affected by the orientation of CBCT images, but for the current study, reoriented images were utilized [2]. Once the images were reoriented, 3D reconstruction of the lateral cephalogram was done and saved as a JPG. The digitized and calibrated images from the iCAT CBCT machine were transferred in the Nemoceph software, and the X and Y coordinates were determined.

Various relevant peer-reviewed articles were considered (Table 2), 20 difficult-to-trace landmarks were then selected (Table 3), and their reliability and reproducibility were checked.

The digitized and calibrated images from Planmeca (a digital cephalometric machine) and iCAT CBCT were transferred to the Nemoceph software, and the X and Y coordinates were determined. The coordinates for each point were traced separately by three different orthodontists (OBA, OBB, OBC) who were designated as observers to check the variability of the points. Intra-observer differences could be due to the nature of the cephalometric landmark, the image quality, and the blurring of the anatomic structures. In contrast, inter-observer differences might be caused by variations in the observer’s training and experience [35].

### 2.3. Blinding

For the prevention of bias, the coordinator created datasets (digital cephalogram and CBCT-generated cephalograms) that were kept in one location and renamed from 1 to 62. All the cephalogram tracings were done two times consecutively by three different observers with a minimum gap of 10 days to eliminate the observer bias in between and to check for the inter-observer variability. Once the tracing was performed, the same coordinator decoded the data into a digital and CBCT-derived cephalogram. These findings were transferred to the excel sheet and subjected to statistical analysis.

### 2.4. Statistical Analysis

The statistical software SPSS version 24.0 was used for the analysis, to compare the findings of the three observers. The centroid for each landmark was obtained by marking the coordinates of the mean by all three observers. For the statistical analysis, mean distance (MD), mean deviations from the centroid, and standard deviations were computed. The gold standard observed in the study was the centroid of the markings by the three observers for each landmark. The error in the detection of each landmark was represented by the mean of the distances from the centroid of the observers’ markings to each observer’s marking. To assess the variation in each axis of all landmarks, the mean deviation was computed. The variations among all the samples of each landmark were represented by standard deviations. To illustrate the distribution of errors over the number of samples, the standard deviations were computed for mean deviation. Statistical analysis was performed by using descriptive and inferential statistics with paired *t*-tests to compare the differences measured by the two methods. Real-scale data are presented in mean ± SD. A *p*-value less than 0.05 was considered as significant at the 95% confidence level.

Step 1: The average of the two readings for each observer was calculated separately for the digital and CBCT-derived cephalograms.

OBA = (Lax + Lax)/2; OBB = (LBx + LBx)/2; OBC = (LCx + LCx)/2:

OA = (Lay + Lay)/2; OB = (LBy + LBy)/2; OC = (LCy + LCy)/2

where x and y are the coordinates and A, B, and C are the observers.

Step 2: The centroid was calculated for both coordinates by using the formula:

Centroid CLx = ((OAx + OBx + OCx)/3; CLy = (OAy + OBy + OCy))/3

Step 3: Calculation of the distance for each observer from the centroid for all three observers.

DLA = √(CLxA − OxA)^2 + (CLyA − OyA)^2,

DLB = √(CLxB − OxB)^2 + (CLyB − OyB)^2 and

DLC = √(CLxC − OxC)^2 + (CLyC − OyC)^2

Step 4: The mean distance was calculated with

MD = (DLA + DLB + DLC)/3

where DL is the distance of the landmark for each observer from the centroid (for observer A (OBA), B (OBB), and C (OBC)) and L is any of the 20 landmarks.

Step 5: The mean Distance was calculated for all 20 landmarks in all 20 datasets. This whole process was adopted for each landmark.

## 3. Results

**Figure 1:**
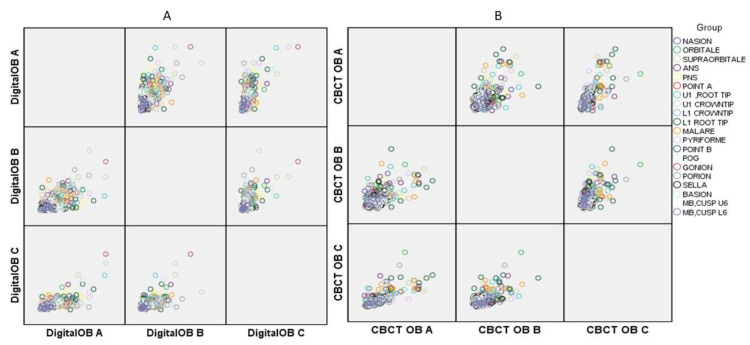
Graph showing associations in the observations taken by three observers in (**A**) Digital (**B**) cone beam computed tomography (CBCT).

**Figure 2:**
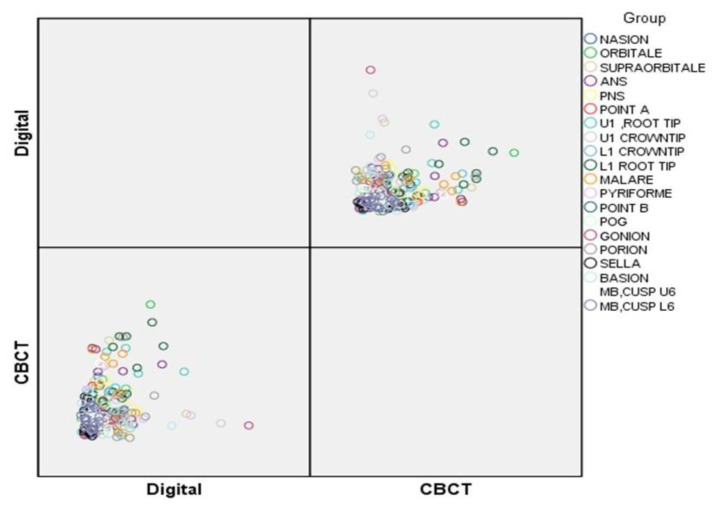
Inter-observer comparison of the plotting of the landmarks between the Digital and CBCT images.

Landmark identification is difficult, especially in patients with a cleft lip and palate. The inter-observer variability of the 20 difficult-to-trace landmarks selected presented an excellent correlation (0.988). The mean distance of the digital and CBCT-derived cephalograms was recorded for all three observers along with the overall mean of the same. A comparison of landmark identification on the digital and CBCT-derived cephalograms was also performed using the *t*-test, and the *p*-value was calculated (a *p*-value less than 0.05 is considered significant at the 95% confidence level and a *p*-value less than 0.01 is significant at the 99% confidence level). The inter-observer findings between observers (OA, OB, and OC) for the digital cephalogram and CBCT-derived cephalograms are presented in Table 4 and Figure 1 and Figure 2. When comparing the digital cephalogram and CBCT-generated cephalograms, the plotting of points PNS (*p* < 0.05), ANS (*p* < 0.01), upper 1 root tip (*p* < 0.05), lower 1 root tip (*p* < 0.05), malare (*p* < 0.05), pyriforme (*p* < 0.05), porion (*p* < 0.01), and basion (*p* < 0.05) was statistically significant.

## 4. Discussion

Cleft lip and/or palate (CL ± P) malformations are the most common congenital abnormalities in the craniofacial region and present a severe problem for health delivery systems throughout the world. Depending on the ethnicity and geographic location of the population, wide variations in the prevalence of cleft lip and palate have been reported, with higher rates amongst Asians and American Indians (one in 500 births). An isolated cleft palate is more frequently found in females than in males, at a ratio of 2:1. In contrast, there is a 2:1 male-to-female ratio for cleft lip with or without cleft palate [36].

Owing to their knowledge of the craniofacial complex and their expertise in tooth movement and dentofacial management, orthodontists’ role in the cleft palate team is indispensable. Orthodontic treatment can have a dental effect, orthopedic reverberations, or both [37], but a prerequisite for successful orthodontic management is an understanding of the site, extent, and severity of the cleft-related craniofacial dysmorphology. The advancements in technology and the incorporation of novel methods aid in striving toward an accurate diagnosis. Researchers have worked diligently to achieve a micro-level accuracy in landmark identification, even comparing the efficiency in the projection of different CBCT machines [31] with a deluge of articles on the same. However, there is a paucity of data on landmark repeatability and reproducibility in the craniofacial morphologies of CL ± P patients, which is challenging to accomplish. Hence, the present study was undertaken in CL ± P patients to decipher the landmarks and verify their reproducibility so these can be applied clinically and aid in treatment planning.

The earlier studies regarding CL ± P and their analysis made use of cephalograms alone, as that was the only imaging modality available at the time. Hotz and Gnoinski [7] advised that caution should be used in the interpretation of results involving point PNS and point A, whereas Mølsted et al. [8] reported errors for the skeletal variables like point A, ANS, and PNS. The ANS and PNS points were both statistically significant in the present study.

The Nasion and Orbitale points were not significant in our study, which is in accordance with Grauer D et al. [28] who observed similar results, whereas Chang ZC et al. [29] implied that the identification error increased at the Nasion and Orbitale on the CBCT-derived cephalograms. Ludlow JB et al. [21] concluded that the Nasion and Orbitale had no significant variation in anterior–posterior (AP) measurement but had a significant variation of caudal cranial (CC) values. Moreover, Chien PC et al. [3] also presented no variation in the Nasion in inter and intra-observer variation, whereas significant inter-observer variation was perceived in the Orbitale. Lagravère MO et al. [38] concluded that the mean difference for the Nasion was not high, but the Orbitale showed a high mean difference. The landmarks of Gonion, Nasion, Orbitale, and Anterior Nasal Spine (ANS) showed the most considerable median Euclidean distances for both intra- and inter-observer measurements according to Katkar et al. 2013 [31]. According to Durao APR et al., the Gnathion (Gn) point was the least reliable landmark for orthodontists, while the Orbitale (Or) was the least reliable landmark for dentomaxillofacial radiologists [32].

The supraorbitale point presented with statistically non-significant values, however. Turhan-Haktanir N et al. [39] studied records of 399 patients on computed tomography (CT) and concluded that the presence of a double foramen/notch was higher on the right side as cephalograms are usually taken from the right. Moreover, various types of supraorbital transcranial exits were observed by Kyu LN 2019 [40], of which the frontal notch was the most common. Woo SW et al. [41] also revealed the variation and characteristics of the supraorbital foramen or notch in a Korean population based on 3D-CT images. These findings were in contrast to those of the present study.

The anterior nasal spine (*p* ≤ 0.05) point was significantly variable in inter-observer tracings. Similar findings were made by Ludlow JB et al. [21], Grauer D et al. [28], McClure SR 2005 [27], and Durao APR [32], wherein the ANS showed significant variation along with point A [6,21,27], Condylion [28,32], and Gnathion [21,33]. Conventional landmarks such as points A, ANS, and PNS are challenging to mark in children with unilateral cleft lip and palate (UCLP). However, in a study by Bongaarts CA [6], no other landmarks were found to be easier to trace. Care should be taken while interpreting the results of cephalometric studies in UCLP patients, due to inter-observer tracings, digitization, and, therefore, measurement errors utilizing landmarks like points A, ANS, and PNS [6]. Chang ZC et al. [29] also showed a positive correlation, implying that the identification error increased at the ANS on the CBCT-derived cephalograms. An intraclass correlation coefficient (ICC) of more than 15% was found by Chien PC et al. [3], who showed significant variation in the inter-examiner evaluation.

Due to a significant shift from the midline in the premaxillary region of young patients, a variation in the ANS can be explained [42]. Point PNS can be influenced by a possible palatal shelf rotation from the centerline and/or a deficiency in the size of the posterior palate [42].

The posterior nasal spine (*p* ≤ 0.05) presented with significant inter-observer tracings. Chang ZC et al. [29] showed an increased error in the landmark identification of the PNS. Bonagaarts CA [6] along with Ludlow JB et al. [21] and Ghoneima A [34] also presented significant variations. However, no variation in the PNS was found in a study by Chien PC et al. [3]. Lagravère MO et al. [38] also inferred the difference in the plotting of point PNS in the X-axis for both the inter- and intra-examiner evaluation.

The root tip of the maxillary and the mandibular incisor showed significant variability. These findings are in agreement with that of Chien PC et al. [3], who had a significant variation for the lower incisor root tip, but no significant finding for the upper incisor roots. Similar observations were reported by Chang ZC et al. [29], with significant findings in the root apex of the maxillary and the mandibular incisor root apex. The reason for the difficulty of root tip identification can be attributed to tooth germ moulding, unerupted teeth in the anterior part of the maxilla, and a reduced radio-opacity due to the presence of cleft [7]. Our study shows no significant finding for incisor tips. However, Grauer D et al. [28] also showed a significant variation in the maxillary incisor tip. Additionally, Ludlow JB et al. [21] and Ghoneima A [34] presented significant findings on the maxillary and mandibular incisor tips.

In the present study, the points malare and pyriforme had a significant discrepancy. These findings are in conjugation with Suri S et al. [37], who measured the asymmetry of these two points using axial and transverse CT slices and noted no significant measurement for the malare, but a significant reduction in the sagittal positioning of the pyriforme on the cleft side.

Significant variability was observed in bilateral landmarks like the porion, along with the basion. Chang ZC et al. [29] and Ghoneima A [34] showed that there was a positive regression correlation for the basion with a high variability, but no significance for the gonion, which was found to be significant in studies conducted by Ludlow JB et al. [21], Katkar RA et al. [31], and Park J et al. [20]. The variation in the porion can be attributed to the difference in opinion in localizing the point. Some authors consider the porion as a landmark in the soft tissues of the ear canal, whereas others feel it is on a bone/soft-tissue margin. A high variability was observed by Chang ZC et al. [29] and Durao APR [32]. Chien PC et al. [3] revealed a very high degree of variability for the basion for both the intra- and inter-observer evaluation, whereas the porion and gonion showed a significant variation in the Y-axis for inter-observer variability.

Since no study was available for comparing the landmark identification errors in patients with CL ± P, the comparison was made with present studies published on the general population. Many articles have been published concerning the use of CBCT in patients with a cleft lip and/or palate (CL ± P). Now, CBCT has been proven to be a better choice for assessing bone volume, deficiencies, and root development, because it provides a better image quality at a significantly lower radiation dose. However, no evidence is available, showing that CBCT is more informative than 2D concerning facial soft-tissue analysis in patients with CLP.

CBCT scans should be carried out while weighing the pros and cons. It can be regarded as a highly reliable diagnostic tool in both simple and complex cases with CLP where 3D assessment is mandatory for making the most appropriate therapeutic decision.

## 5. Limitations

The primary limitation can be attributed to the non-inclusion of bilateral cleft lip and palate patients, who also constitute a large proportion of patients with a cleft lip and palate. The identification of landmarks in cases of cleft lip and palate is arduous, and not all observers are able to localize the points in a similar manner. In the present study, accuracy may have been affected by the operator’s skills, expertise, and exhaustion due to frequently repeated measurements. In addition, the present study was a retrospective analysis; therefore, clinical examination of the study sample could not be done and relied solely on the medical records and data.

## 6. Conclusions

1. The CBCT images provide a significantly more precise location of the anterior nasal spine, Point A, posterior nasal spine, pyriforme, and malare, overcoming the problem caused due to clefting in the nasomaxillary complex.
2. Bilateral landmarks like the porion, gonion, and basion were also significantly more precise in CBCT images when compared with digital cephalograms.

## Figures and Tables

**Table 1:** Sample Characteristics.

	Boys (*n* = 18)			Girls (*n* = 13)			Total Sample (*n* = 31)		
	Mean	SD	Range	Mean	SD	Range	Mean	SD	Range
Age (yrs.)	12.035	0.690	10–14	12.13	0.724	10–14	12.09	0.698	10–14

**Table 2:** Studies reviewed to determine difficult-to-trace landmarks.

S.No.	Author (Year)	Difficult-to-Trace Points in These Studies/Significant Findings
1.	Bongaarts CA et al., 2008 [6]	Point A, ANS, and PNS
2.	Ludlow JB et al., 2009 [21]	Anterior Posterior (AP): Point A, ANS, Point B, Go, Mandibular Incisor Tip
		Cephalocaudal (CC): ANS, Co, Go, Na, Or
		Avg AP and CC: Point A, ANS, Co, Go, Mandibular Incisor Tip, Me, Na
3.	Chien PC et al., 2009 [3]	INTER-OBSERVER RELIABILITY
		X Coordinate: Subspinale, ANS, Ba, Co, L1 Root, sigmoid notch
		Y Coordiate: Ba, co, go, L1tip, Or, Po, S, Sigmoid notch, U1 tip
		INTRA-OBSERVER RELIABILITY
		X coordinate: Ba
		Y coordinate: Ba, L1 tip, Me, Or, Sella, Sigmoid Notch
4.	Grauer D et al., 2010 [28]	ANS, U1tip, Point B
5.	Chang ZC et al., 2011 [29]	Intercept: S, Or, ANS, Point A, L1, L1R, Point B, Me, Go, Ba, PNS
		Interaction with CBCT mode: N, Gn, Me, Ba.
6.	Zamora N et al., 2012 [30]	Supraorbitale, Right zygion, PNS
7.	Katkar RA et al., 2013 [31]	Go, Na, Or, ANS and Md1 root.
8.	Durao APR et al., 2015 [32]	X coordinate: Or, Po, Go, Co, PNS
		Y coordinate: Co, Point B, Point A
9.	Neiva MB et al., 2016 [33]	Right Co, Gn, Left ramus point, Right and left zygomatic suture
10.	Ghoneima A et al., 2017 [34]	X coordinate: Ba, Point B, Me, U1, L1
		Y coordinate: Ba, PNS.
11	Park J et al., 2019 [20]	Skeletal: Bilateral structures showed more errors than midline structures. Go was least reliable.

Point A, Point B, Anterior Nasal Spine (ANS), Condylion (Co), Posterior Nasal Spine (PNS), Gonion (Go), Mandibular Incisor Tip (L1), Maxillary incisal tip (U1), Nasion (Na), Oribitale (Or), Menton (Me), Supspinale, Sigmond notch, Porion (Po), Sella (S), Basion (Ba), Gnathion (Gn), Supraorbitale, Zygion, Zygomatic suture.

**Table 3:** Twenty difficult-to-trace landmarks.

S.No.	Landmark	Definition of Landmark
1.	Nasion	Intersection of the internasal suture with the nasofrontal suture in the midsagittal plane.
2.	Orbitale	Most inferior point on the infraorbital rim
3.	Supra orbitale	Most superior point on the infraorbital rim
4.	ANS	Tip of the anterior nasal spine
5.	PNS	Point along the palate immediately inferior to the pterygomaxillary fossa
6.	Point A	Deepest point of the curve of the maxilla, between the anterior nasal spine and the dental alveolus
7.	U1, root tip	Root tip of the maxillary incisor
8.	U1, crown tip	Incisal tip of the maxillary incisor
9.	L1, crown tip	Incisal tip of the mandibular incisor
10.	L1, root tip	Root tip of the mandibular incisor
11.	Malare	Most prominent anterolateral point on the zygomatic bone that lends the malar prominence to the face
12.	Pyriforme	Anterolateral margin of the nasal aperture represented by the most anteromedial point on the maxilla, forming the bony alar base
13.	Point B	The deepest midline point on the mandible between the infradentale and the pogonion
14.	Pog	Most anterior point on the midsagittal symphysis
15.	Gonion	Point along the angle of the mandible, midway between the lower border of the mandible posterior ascending ramus
16.	Porion	Most superior point of the right external auditory meatus
17.	Sella	Center of the pituitary fossa of the sphenoid bone
18.	Basion	Most inferior point on the anterior margin of the foramen magnum, at the base of the clivus
19.	MB cusp, U6	Mesio-buccal cusp of the maxillary first molar
20.	MB cusp, L6	Mesio-buccal cusp of the mandibular first molar

**Table 4:** Comparison of observations obtained by different observers for digital and CBCT derived cephalograms using *t* test.

Landmarks	Comparisons (Paired *t*-Test) *p*-Value									
	Digital			CBCT			Digital vs CBCT			
	A VS B	A VS C	B VS C	A VS B	A VS C	B VS C	A	B	C	OVERALL
NASION	0.362	0.520	0.093	0.112	0.713	0.060	0.366	0.732	0.460	0.841
ORBITALE	0.191	0.417	0.861	0.119	0.133	0.773	0.972	0.481	0.184	0.910
SUPRAORBITALE	0.974	0.235	0.118	0.005	0.129	0.157	0.913	0.041	0.167	0.177
PNS	0.220	0.113	0.009	0.057	0.196	0.375	0.120	0.153	0.001	0.016 *
ANS	0.115	0.111	<0.001	0.010	0.151	0.010	0.018	0.006	<0.001	<0.001 *
POINT A	0.225	0.395	0.006	0.029	0.709	0.028	0.599	0.859	0.023	0.600
U1 ROOT TIP	0.028	0.678	0.037	0.033	0.238	0.109	0.095	0.119	0.038	0.039 *
U1 CROWNTIP	0.003	0.843	0.182	0.144	0.176	0.270	0.088	0.158	0.246	0.162
L1 CROWNTIP	0.027	0.100	0.225	0.221	0.174	0.063	0.799	0.828	0.947	0.995
L1 ROOT TIP	0.052	0.761	0.099	0.031	0.246	0.025	0.067	0.353	0.017	0.013 *
MALARE	0.384	0.226	0.001	0.028	0.027	0.317	0.277	0.004	0.635	0.043 *
PYRIFORME	0.793	0.237	0.173	0.013	0.648	0.007	0.103	0.021	0.002	0.015 *
POINT B	0.337	0.080	0.716	0.236	0.271	0.773	0.680	0.508	0.410	0.894
POG	0.003	0.568	0.014	0.097	0.496	0.164	0.074	0.678	0.040	0.069
GONION	0.323	0.337	0.120	0.061	0.643	0.039	0.127	0.143	0.149	0.136
PORION	0.352	0.041	0.028	0.318	0.455	0.962	<0.001	<0.001	0.003	<0.001 *
SELLA	0.002	0.002	0.961	0.138	0.994	0.055	0.594	0.196	0.092	0.183
BASION	0.606	0.732	0.973	0.027	0.506	0.022	0.037	0.011	0.026	0.011 *
MB, CUSP U6	0.008	0.008	0.416	0.061	0.037	0.035	0.528	0.302	0.765	0.483
MB, CUSP L6	0.012	0.576	0.036	0.523	0.280	0.153	0.403	0.219	0.112	0.436

* *p*-value < 0.05 is considered as significant.

## Data Availability

The data presented in this study are available on request from the corresponding author.
